# Influence of Second-Trimester Ultrasound Markers for Down Syndrome in Pregnant Women of Advanced Maternal Age

**DOI:** 10.1155/2014/785730

**Published:** 2014-03-25

**Authors:** Mariza Rumi Kataguiri, Edward Araujo Júnior, Luiz Claudio Silva Bussamra, Luciano Marcondes Machado Nardozza, Antonio Fernandes Moron

**Affiliations:** Department of Obstetrics, Paulista School of Medicine—Federal University of São Paulo (EPM-UNIFESP), Rua Carlos Weber, 956, apto. 113 Visage, Vila Leopoldina, 05303-000 São Paulo, Brazil

## Abstract

The objective of the present study was to evaluate the influence of second-trimester ultrasound markers on the incidence of Down syndrome among pregnant women of advanced maternal age. This was a retrospective cohort study on 889 singleton pregnancies between the 14th and 30th weeks, with maternal age ≥ 35 years, which would undergo genetic amniocentesis. The second-trimester ultrasound assessed the following markers: increased nuchal fold thickness, cardiac hyperechogenic focus, mild ventriculomegaly, choroid plexus cysts, uni- or bilateral renal pyelectasis, intestinal hyperechogenicity, single umbilical artery, short femur and humerus length, hand/foot alterations, structural fetal malformation, and congenital heart disease. To investigate differences between the groups with and without markers, nonparametric tests consisting of the chi-square test or Fisher's exact test were used. Moreover, odds ratios with their respective 95% confidence intervals were calculated. Out of the 889 pregnant women, 131 (17.3%) presented markers and 758 (82.7%) did not present markers on the second-trimester ultrasound. Increased nuchal fold (P < 0.001) and structural malformation (P < 0.001) were the markers most associated with Down syndrome. The presence of one marker increased the relative risk 10.5-fold, while the presence of two or more markers increased the risk 13.5-fold. The presence of markers on the second-trimester ultrasound, especially thickened nuchal fold and structural malformation, increased the risk of Down syndrome among pregnant women with advanced maternal age.

## 1. Introduction

Although chromosomal abnormalities occur at low frequency in the population, around 0.5% to 2% [1], they contribute significantly to increased perinatal morbidity and mortality [2]. Trisomy is the most frequent chromosomal abnormality, especially of chromosome 21, that is, Down syndrome. Since Down syndrome is difficult to diagnose during the prenatal period and because there is the possibility of survival after birth, it contributes to increasing the statistics of cases of mental retardation. Thus, prenatal screening is important, especially among women of advanced maternal age, that is, greater than or equal to 35 years [3].

The presence of certain alterations on the second-trimester ultrasound, called markers, enables increased sensitivity in screening for trisomy 21. This can reach up to 84% and possibly surpass 90% when heart markers are included [4–7].

The challenge is to distinguish the presence of these small alterations on the second-trimester ultrasound, between chromosomally abnormal and normal fetuses, considering that the latter may also present these markers at a rate of around 13% to 17%, which can be considered to be a high percentage of false positives [8]. The importance of this challenge is greater among pregnant women of advanced maternal age, when the relationship with Down syndrome becomes closer [9]. If, on the one hand, these markers enable good detection of aneuploidy, on the other, they lead to an unacceptably high rate of invasive procedures, thereby exposing chromosomally normal fetuses to a risk of death or unnecessary complications [10]. Thus, second-trimester ultrasound can be used as a method to assist in evaluating the risk of fetal aneuploidy, thereby making it possible for the couple to better evaluate the need for an invasive examination to assess the fetal karyotype [11].

The objective of the present study was to determine associations that might exist between some second-trimester ultrasound markers and Down syndrome, among pregnant women of advance maternal age, who underwent fetal chromosomal analysis through amniocentesis.

## 2. Materials and Methods

This was a retrospective cohort study on 889 singleton pregnant women aged ≥ 35 years, who underwent genetic amniocentesis. The present study was approved by the Research Ethics Committee of the Federal University of São Paulo (UNIFESP). All the patients who voluntarily agreed to participate signed a consent form.

The present study was carried out at the Department of Obstetrics of UNIFESP and at Santa Joana Hospital, São Paulo, SP, Brazil. Singleton pregnancies between their 14th and 30th weeks that would undergo genetic amniocentesis because of maternal age ≥ 35 years, with or without markers for Down syndrome on the second-trimester ultrasound, were selected. Pregnant women with a history of genital bleeding over the past seven days, suspected premature rupture of membranes, fetal heart rate abnormalities, suspected congenital infection (from ultrasound alterations), or use of potentially teratogenic drugs were excluded.

The second-trimester ultrasound was performed before the genetic amniocentesis, using the Logic 500 device (General Electric Medical System, Milwaukee, WI, USA) equipped with a convex transducer (3–5 MHz). In this examination, the presence of the following markers was evaluated: increased nuchal fold thickness (≥6 mm), mild ventriculomegaly (atrium of the lateral ventricle measuring between 12 and 15 mm), choroid plexus cysts, cardiac hyperechogenic focus, unilateral or bilateral renal pyelectasis (renal pelvis measuring ≥4 mm between the 15th and 20th weeks and ≥5 mm between the 21st and 30th weeks), intestinal hyperechogenicity, single umbilical artery, short femur length (observed/expected measurement < 0.90), short humerus length (observed/expected measurement < 0.89), alterations at the extremities such as the shape or position of hands or feet, structural malformations, or congenital heart diseases.

To carry out the amniocentesis, a 20 mL syringe and a 20 G needle were used after applying local anesthesia consisting of 2% lidocaine. 20 mL of clear liquid were collected, without blood contamination, and were immediately forwarded to the cytogenetic laboratory. Pregnant women with a negative Rh blood type, negative indirect Coombs test, or a Rh-positive partner received anti-D immunoglobulin within 72 hours after the procedure, so as to avoid maternal sensitization.

The data were transferred to a worksheet in the Excel 2003 software (Microsoft Corp., Redmond, WA, USA) and were analyzed using the SPSS version 13.0 software for Windows (SPSS Inc., Chicago, IL, USA). Probability calculations were used to search for any relationships between fetal malformations and/or second-trimester ultrasound markers for detecting Down syndrome in women of advanced maternal age, so as to observe whether there were any associations between the presence of markers (singly or in combination) and the presence of Down syndrome and, for each of the markers found, the association of each type of marker with the syndrome. Initially, a descriptive analysis was performed, presenting the data as absolute frequencies (*N*) and relative frequencies (%) for each category of the qualitative variables of the patients' profile. For the quantitative variables, means, standard deviations, and minimum and maximum values were calculated. To investigate possible associations between pairs of characteristics or qualitative variables, nonparametric tests consisting of the chi-square test or Fisher's exact test when necessary were used, with a significance level of 5%. Combined associations of the variables were assessed in a multivariate manner, by means of correspondence analysis. The relative risk calculations and odds ratios were considered significant when the data were within the 95% confidence interval.

## 3. Results

The maternal age ranged from 35 to 47 years, with a mean of 38.8 ± 2.9 years for the 31 pregnant women with fetuses with Down syndrome and 38.6 ± 2.6 years for the 858 pregnant women with chromosomally normal fetuses, without any statistical difference. Similarly, gestational age at the time of amniocentesis did not present any significant difference between the two groups, with a mean of 17.7 ± 3.1 weeks for the Down syndrome cases and 17.3 ± 2.4 weeks for the chromosomally normal fetuses.

Among the 889 pregnant women, 131 (17.3%) presented markers and 758 (82.7%) did not present markers on the second-trimester ultrasound.

Table 1 presents the distribution of the 31 fetuses with Down syndrome and their correlation with the ultrasound markers, which presented a statistically significant difference (*P* < 0.001).

Table 2 presents the distribution of the Down syndrome cases, between the two groups studied, with and without markers, according to maternal age, in which there was a statistically significant difference for the maternal ages of 37, 38, and ≥40 years. The odds ratio calculation did not show any increased occurrence of Down syndrome among women ≥40 years of age in the presence of ultrasound markers (OR = 1.74; 95% CI: 0.84–3.57).

Table 3 presents the correlation between some second-trimester ultrasound markers and Down syndrome, with positive correlations with thickened nuchal fold, short femur, and structural malformations.  Table 4 presents the odds ratios for some ultrasound markers in relation to Down syndrome, showing increased occurrence of this syndrome in the presence of thickened nuchal fold and short femur length.  Table 5 presents the sensitivity, specificity, and positive and negative predictive values for the ultrasound markers in occurrences of Down syndrome.

Table 6 presents the calculation of chances between the number of ultrasound markers and Down syndrome. We observed that the greater the number of markers was, the higher the chance of Down syndrome was.

## 4. Discussion

Amniocentesis has traditionally been offered during the second trimester, to patients with a high risk of Down syndrome, that is, pregnant women with advanced maternal age. However, using only this parameter for screening, only around 31% of the cases of trisomy 21 are diagnosed, with false positive rates of 13% to 14% [12]. Investigation of aneuploidy markers on second-trimester ultrasound presents high rates of false positives (12% to 15%) due to the high frequency of these markers in low-risk populations and, especially, due to the low sensitivity when these markers are present separately [13]. Therefore, it is important to define and study this type of population separately, so as to achieve screening of greater reliability, with better results for detecting chromosomal abnormalities [10].

It has been suggested that a normal second-trimester ultrasound examination reduces the initial risk of Down syndrome by 50% to 80% [11, 14, 15]. The present study obtained a similar result, in which there was a 90% decrease in risk in the absence of ultrasound markers and/or structural malformations.

According to Nyberg et al. [8], in the presence of at least one marker, the risk of Down syndrome increased two-fold from the initial risk; in the presence of two markers, the risk increased tenfold and, when three or more markers were present, the risk increased more than a hundredfold. On the other hand, while Benacerraf [16] also showed that in the presence of one marker the risk increased twofold, they showed that in the presence of two or more markers, the risk increased 23-fold. In the present study, in the presence of one marker, the risk increased 10.5-fold and when two or more markers were present, the risk increased 13.5-fold.

Increased nuchal fold (≥6 mm) is without doubt the marker most correlated with Down syndrome during the second trimester, and it is present in approximately 39% to 45% of the cases [16]. In the present study, we observed nuchal fold sensitivity of 39% with specificity of 96%, which was statistically significant. Although specificity of this marker was similar to that found by DeVore [17] and Aagaard-Tillery et al. [18] (99.2%, 99%, resp.) the same did not occur with sensitivity (28.8% and 18%, resp.). The variability of specificity may be accompanied by an even greater variability in likelihood ratio (LLR) with these previous studies finding rates of 53.4 and 49, respectively, whereas the present study determined a LLR of 15. Other studies, on the other hand, showed LLR varying from 10 to 17 [8, 15, 19].

The presence of structural malformation has, over the years, remained a good parameter for diagnosing Down syndrome [19]. In the present study, structural abnormalities showed sensitivity of 19%, with specificity of 97%. The most frequent type of malformation in the syndrome cases was cystic hygroma (50%). These results are in agreement with those reported by other authors, such as Sohl et al. [20] and Bromley et al. [15], who described structural malformations presenting sensitivities of 16.4% to 19.5% in screening for this syndrome, respectively.

It seems that shorter femur is not a good marker for Down aneuploidy. Shorter femur was shown by just 1 out of the 31 of Down cases. Furthermore, previous studies report a high frequency of this marker among euploid individuals [21, 22]. In the present study, the risk increased 14-fold, with a very wide confidence interval, reflecting in low accuracy. Similarly, Cho et al. [23] observed that short femur length was a poor marker for Down syndrome in a Korean population.

The intracardiac hyperechogenic focus is a controversial marker, because it is a common finding among the normal population during the second trimester [24]. Other studies have correlated this marker with Down syndrome in proportions of 16% to 18% [25, 26]. In the present study, this marker was present in 10.3% of the chromosomally altered cases. Most were present together with other ultrasound markers, thus presenting a statistically significant tendency towards an association with increased risk. However, when present separately, this marker did not present any significant association with Down syndrome.

The congenital heart disease (CHD) was present in one fetus with Down syndrome (1/31 = 3.2%) and in two euploid fetuses (2/858 = 0.23%). We included the cases of CHD in the group of structural malformations, because the number cases were insufficient to correlate them with the Down syndrome. It has occurred because of the low gestational age at the moment of ultrasound exam (17 weeks), when the CHD rate detection is smaller than after 20 weeks [27]. Furthermore, the ultrasound exams were realized by sonographers without expertise in fetal echocardiography, justifying the low rate detection. The main structural CHD in fetuses with Down syndrome is atrioventricular septal defect [28]; however, the detection rate of this CHD during prenatal ultrasound is low [29].

In the present study, we performed a statistical calculation comparing the diagnosis of Down syndrome in the presence and absence of ultrasound markers. In considering the calculation with two markers, the sensitivity reduced markedly to 29.4%, but with an increase in specificity to 97%. Thus, we considered that second-trimester ultrasound had a high chance of screening for Down syndrome in the presence of at least two markers in the population of pregnant women with advanced maternal age.

The use of ultrasound markers during the second trimester to screen for Down syndrome is subject to bias, since there are still no definitive studies regarding the sensitivity and specificity of each marker or set of markers [30]. However, through appropriate standardization of the types of populations and the studies carried out, the analyses on these markers will become better, because the interactions of each marker, separately or in association with other markers, will be evaluated, thereby resulting in a second-trimester screening for Down syndrome that is more reliable overall [31].

The accuracy of ultrasound as Down syndrome screening examination has been improved by combining its findings in the first trimester of pregnancy with biochemical screening in the first and second trimester (FASTER trial). Currently, a much more accurate screening tool is available. Noninvasive prenatal testing (NIPT) is able to detect trisomy 21 directly on free fetus DNA fragments in mother's blood stream. It has high accuracy; however, it is associated with higher costs and equipment availability, which is not universally provided, mainly in developing countries as Brazil. This makes genetic ultrasound a relatively reliable screening tool, thus supporting investigative work undertaken to better use this tool.

## 5. Conclusion

In summary, increased nuchal fold and presence of structural malformation presented significant associations with Down syndrome, especially when associated with other ultrasound markers, among pregnant women of advanced maternal age.

## Figures and Tables

**Table 1:** Description of the cases of Down syndrome according to the groups with and without ultrasound markers (*P* < 0.001).

Group of pregnant women	Down syndrome					
	Present		Absent		Total	
With markers	19	14.6%	112	85.5%	131	100.0%
Without markers	12	1.6%	746	98.4%	758	100.0%

Total	31	3.5%	858	96.5%	889	100.0%

**Table 2:** Distribution of the cases of Down syndrome according to maternal age and ultrasound markers.

Maternal age (years)	With markers		Without markers		Statistical analysis (*P* < 0.05)
	Present	Absent	Present	Absent	
35	4	46	2	89	*P* = 0.11
36	2	19	1	91	*P* = 0.08
37	2	13	1	112	*P* = 0.03
38	2	9	1	101	*P* = 0.02
39	1	8	1	94	*P* = 0.16
≥40	8	17	6	259	*P* < 0.001

Total	19	112	12	746	

**Table 3:** Correlation between ultrasound markers and Down syndrome.

Marker	Down syndrome		Statistical analysis
	Present	Absent	
Nuchal fold thickness ≥ 6 mm	12/31	35/858	*P* < 0.001
Intracardiac hyperechogenic focus	4/31	35/858	*P* = 0.05
Short femur length	1/31	2/858	*P* = 0.10
Structural malformation	6/31	26/858	
Duodenal Atresia	1	5	*P* < 0.001
Esophageal Atresia	1	4	
Cystic Hygroma	3	0	
Meningomyelocele	0	6	
Spina Bifida	0	4	
Congenital heart disease	1	2	
Hydrocephalus	0	5	

**Table 4:** Odds ratios for the ultrasound markers for Down syndrome.

Marker		Down syndrome		Odds ratio	Confidence interval (95%)
** **	** **	Absent	Present		
Nuchal fold thickness ≥ 6 mm	Present	35	12	14.9	6.7–33.0
	Absent	823	19		

Intracardiac hyperechogenic focus	Present	35	4	3.5	1.1–10.5
	Absent	823	27		

Short femur length	Present	2	1	14.2	1.3–161.7
	Absent	856	30		

Structural fetal malformation	Present	26	6	7.7	2.9–20.3
	Absent	832	25		

**Table 5:** Sensitivity, specificity, positive predictive value (PPV), and negative predictive value (NPV) of the ultrasound markers for detecting Down syndrome.

Marker	Sensitivity (%)	Specificity (%)	PPV (%)	NPV (%)
Nuchal fold thickness ≥ 6 mm	38.7	95.9	25.5	97.7
Short femur length	32.3	99.8	33.3	96.6
Intracardiac hyperechogenic focus	12.9	95.9	10.3	96.8
Structural fetal malformation	19.3	97.0	18.8	97.1

**Table 6:** Odds ratios between the number of ultrasound markers and Down syndrome.

Number of markers	Down syndrome				Odds ratio	Confidence interval (95%)
	Present		Absent			
0	12	38.7	746	86.9	0.1	0.0–0.2
1	14	45.2	89	10.4	10.5	4.1–23.4
≥2	5	16.1	23	2.7	13.5	3.8–46.2
